# A comprehensive dataset on two-dimensional noble metals: Theoretical insights into physical properties and metal-support interactions

**DOI:** 10.1016/j.dib.2023.109801

**Published:** 2023-11-10

**Authors:** Ivan Shtepliuk

**Affiliations:** Semiconductor Materials Division, Department of Physics, Chemistry and Biology-IFM, Linköping University, S-58183 Linköping, Sweden

**Keywords:** 2D noble metals, Silicon carbide, Surface reconstruction, Epitaxial graphene, Density functional theory, Charge transfer, Hydrogen adsorption

## Abstract

This paper presents a dataset offering profound insights into the formation and physical properties of two-dimensional (2D) noble metals under various configurations, with a primary focus on their role as catalysts for the hydrogen evolution reaction (HER). These data are of significant value to catalysis researchers, materials scientists, and computational chemists, providing them with a detailed understanding of 2D noble metals' behavior as catalysts and enabling advancements in their respective studies. The dataset, thoughtfully structured and meticulously documented, comprises five primary sections, each housing distinct content and analyses. It offers a comprehensive view of the substrate-mediated stabilization and physical properties of 2D noble metals, including silver (Ag), gold (Au), iridium (Ir), osmium (Os), palladium (Pd), platinum (Pt), rhodium (Rh), and ruthenium (Ru). The substrates utilized include bare Si-face 4H-SiC, buffer layer (BuL), and monolayer epitaxial graphene (MEG). The data collection process involves the use of the SIESTA code for density functional theory (DFT) calculations. The vdW-BH functional is consistently applied in conjunction with a double-ζ polarized (DZP) basis set, known for its reliability in capturing nuanced interactions with noble metals. Parameters such as an energy shift of 200 meV and a force tolerance of 0.02 eV/Å are meticulously configured for accurate results. In-depth structural information, including optimized structures in top and side views and Cartesian coordinates for various substrate-metal configurations, is a central component of the dataset. These structural details are pivotal for comprehending the physical properties of 2D noble metals. Furthermore, the dataset encompasses results from charge density difference (CDD) analyses, including cube files, planar-averaged CDD curves, and 3D CDD maps. These analyses provide essential data for understanding the electronic properties of these materials. The dataset also includes outcomes from charge population analyses utilizing Hirshfeld and Voronoi schemes. These analyses offer insights into structural parameters, Hirshfeld charge magnitudes on 2D metal layers, and various energy-related metrics, further enhancing the dataset's richness. In addition to structural data, the dataset presents atomic structures in top and side views of free-standing and substrate-supported 2D noble metals after hydrogen adsorption, along with corresponding Cartesian coordinates. Gibbs free energy (Δ*G*_H*_) data for hydrogen adsorption on both free-standing and substrate-supported 2D noble metals contribute to the dataset's depth. This meticulously curated dataset not only serves as a valuable resource for researchers exploring the properties and behaviors of 2D noble metals but also holds significant reuse potential. Researchers can employ this dataset to validate their computational methods and models in catalysis research, enhancing the quality and reliability of their simulations. Furthermore, it serves as a possible educational tool, fostering hands-on learning for students and emerging researchers in the field of computational materials science and catalysis, thereby promoting methodological consistency within the scientific community.

Specifications Table

**Table:** 

Subject	Physical sciences, Materials Science
Specific subject area	Surfaces and Interfaces, Computational Materials Science
Data format	Raw, Analyzed
Type of data	Table (.txt files), Image and Figure (.TIFF files), Volumetric data (.cube files), Crystallographic data (.XSF files)
Data collection	Data collection involved the utilization of advanced computational resources provided by the National Supercomputer Center (NSC) at Linköping University. High-resolution images of 2D noble metal configurations were obtained using VESTA, a sophisticated visualization and analysis tool. DFT calculations were performed using SIESTA, a renowned simulation package known for its precision and efficiency. For intricate charge density analyses, GaussView 6 was used to calculate the 3D charge density difference (CDD), and Multiwfn was employed for post-processing to extract essential data. MATLAB was utilized to generate *xy*-plane averaged CDD curves, offering insightful visualizations.
Data source location	Institution: Linköpings universitet (LiU) City/Region: Linköping, Östergötland Country: Sweden
Data accessibility	Repository name: Mendeley Data Data identification number: 10.17632/zdz455s7gd.1 Direct URL to data: https://data.mendeley.com/datasets/zdz455s7gd/1

## 1 Value of the Data

• **Value**: These data hold immense value because they provide comprehensive insights into the electronic and catalytic properties of 2D noble metals under various configurations, directly addressing the primary research hypothesis about their efficiency as catalysts for the hydrogen evolution reaction.
• **Beneficiaries**: Catalysis researchers, materials scientists, and computational chemists can significantly benefit from these data. These experts can use the dataset to gain a deep understanding of 2D noble metals' behavior as catalysts, enabling them to advance their studies and contribute to the field's knowledge base. The presented data can potentially be of interest to machine learning experts. They can use this dataset to train machine learning models for predicting catalytic behavior or designing new catalysts with enhanced performance.
• **Reusability**: Other researchers can readily reuse these data to validate their computational methods and models in catalysis research. By using this dataset as a benchmark, they can ensure the accuracy and reliability of their simulations, enhancing the quality of their work and fostering methodological consistency in the scientific community.
• **Educational Resource**: The dataset serves as a valuable educational tool, facilitating hands-on learning for students and emerging researchers in the realm of computational materials science and catalysis.

## 2 Data Description

The dataset [1], presented in this paper, is a comprehensive resource consisting of five primary root directories, each with distinct content and analyses. These directories provide valuable insights into various aspects of the substrate-mediated stabilization and physical properties of 2D noble metals (Ag, Au, Ir, Os, Pd, Pt, Rh, Ru). Here, a detailed overview of the dataset structure is provided, including its folders, subfolders, and files, with the aim of facilitating clear comprehension by users. Visual aids such as figures and tables are used to present key information without introducing interpretations or conclusions.

**Fig. 1:**
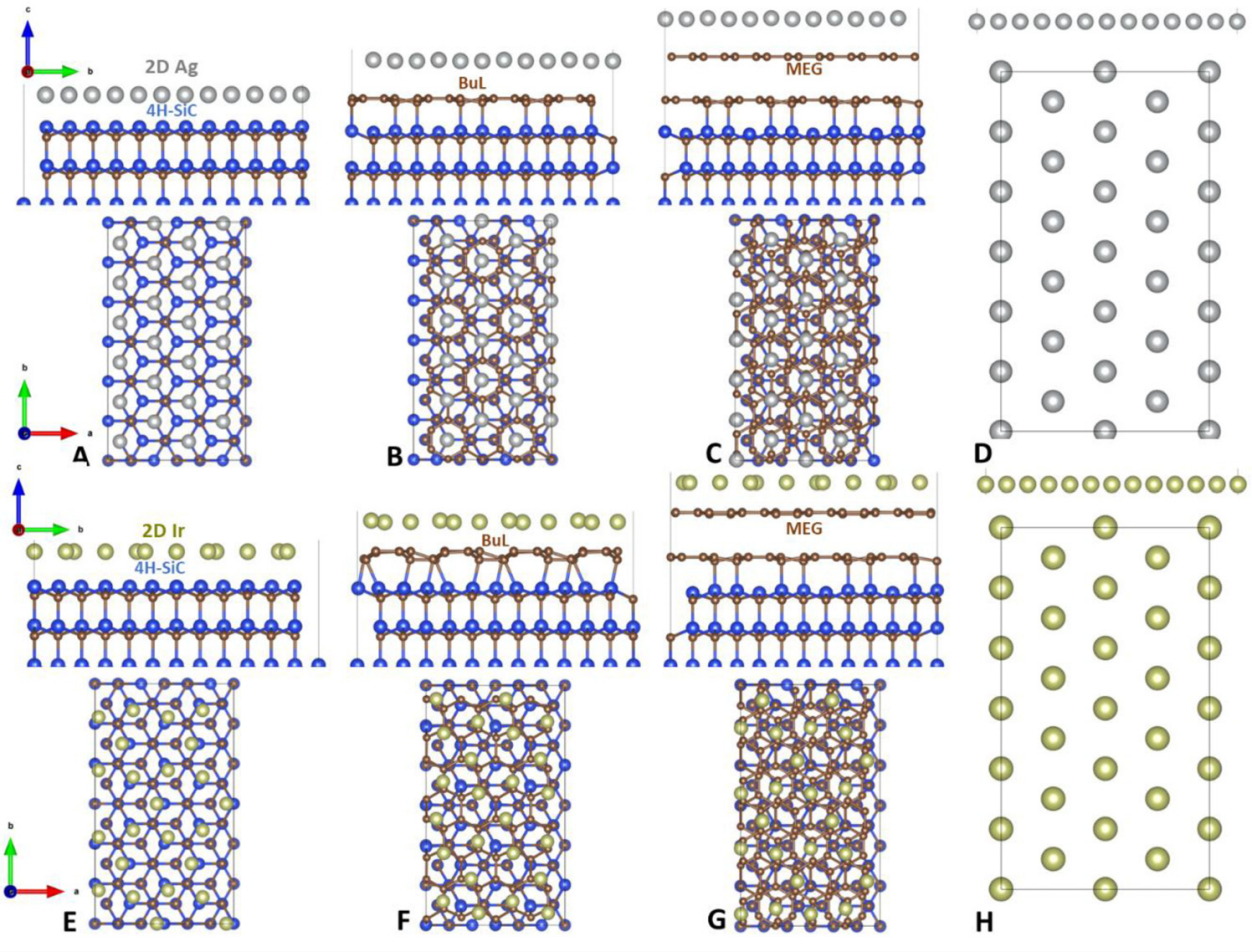
(Side and top views) Optimized atomic structures of substrate-supported (4H-SiC, BuL and MEG) and free-standing Ag (A–D) and Ir (E–H) monolayers, respectively. Blue, brown, gray, and mustard green designate Si, C, Ag, and Ir atoms, respectively.

*“Structure_2Dmetals”* directory encompasses five subfolders, four of which contain eight additional subfolders each. These subfolders meticulously organize information regarding free-standing and substrate-supported 2D noble metals. They include optimized structures, both in top and side views, as well as cartesian coordinates for all substrate-metal configurations. These structural details are essential for understanding the physical properties of these materials. Fig. 1 illustrates top and side views of the optimized structures of free-standing and substrate-supported Ag and Ir monolayers, providing valuable visual context. For more details on other 2D noble metals, readers are referred to this directory. Notably, all cases considered two supercell models of 2D noble metals: (1 × 3 × 1) and (2 × 6 × 1), respectively. The “*Substrates*” folder, found within the “*Structure_2Dmetals*” directory, contains structural data pertaining to the substrates utilized as supports for 2D noble metals. This includes bare 4H-SiC, buffer layer, and epitaxial graphene. The structural information within this directory is crucial for comprehending the interactions between noble metals and their supporting substrates.

**Fig. 2:**
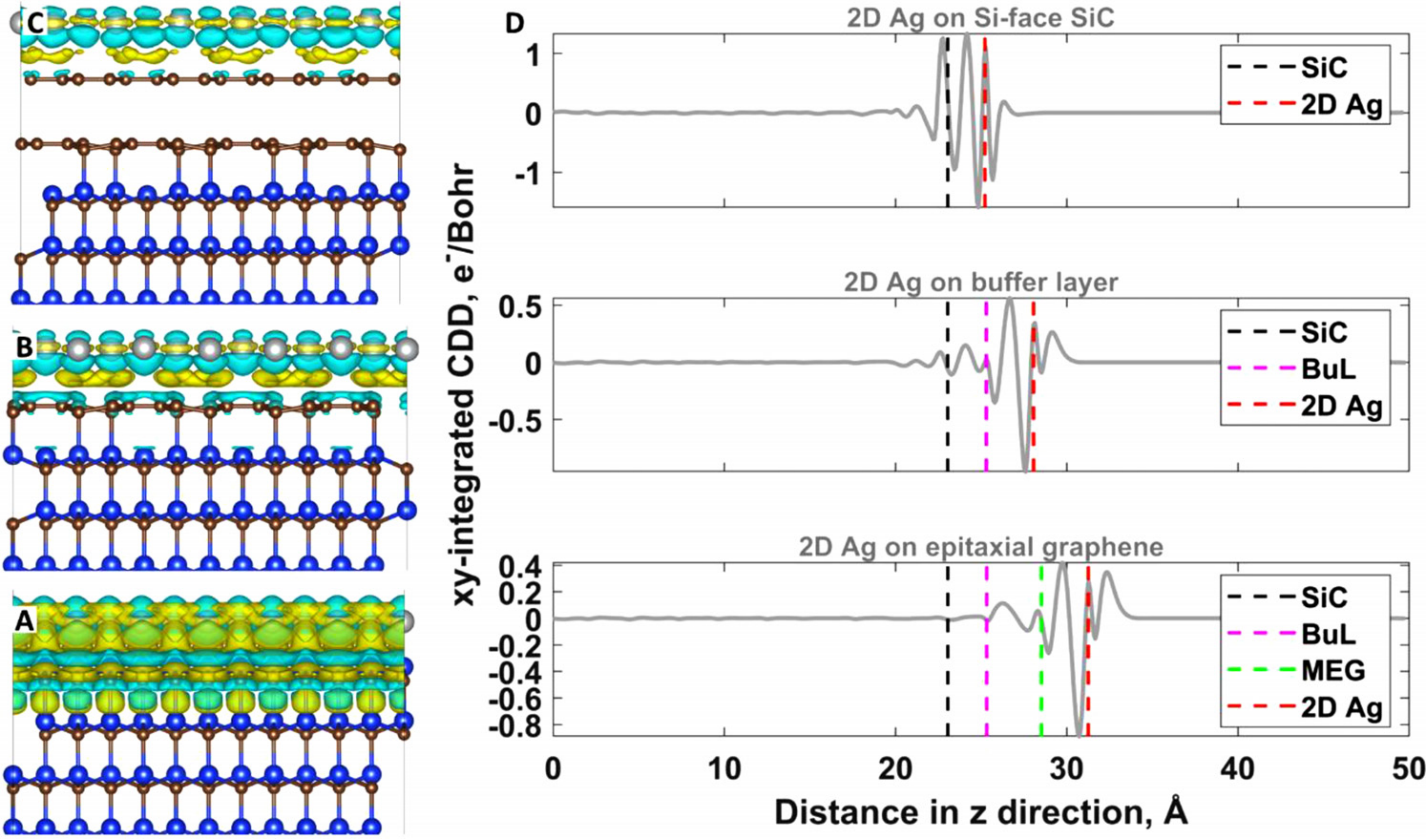
In panels (A), (B), and (C), 3D-CDD maps depict the interfaces of 2D Ag/SiC, 2D Ag/BuL/SiC, and 2D Ag/MEG/BuL/SiC, respectively. Yellow and cyan colors represent positive (charge accumulation) and negative (charge depletion) Δ*ρ*, with an iso-surface level set at 1 × 10^−3^ e·*Å*^−3^. The calculation of CDD is as follows: Δ*ρ* = *ρ*_2DAg/support_ – *ρ*_2DAg_ − *ρ*_support_. In panel (D), the planar-averaged charge density difference for the 2D Ag/SiC, 2D Ag/BuL, and 2D Ag/EG systems is presented as a function of position along the z-direction. The dashed vertical lines denote key locations, including the topmost SiC layer, BuL, graphene, and the 2D Ag layer, respectively. Positive and negative values of the CDD indicate charge accumulation and charge depletion regions, offering insights into the charge distribution at these interfaces.

“*ChargeDensityDifference_2Dmetals*” root directory comprises eight folders, each housing the results of CDD analyses conducted on the considered structures. These analyses encompass cube files, planar-averaged CDD curves, and 3D CDD maps. Fig. 2 showcases the CDD analysis results for substrate-supported 2D silver, serving as a representative case.

“*HirshfeldChargeAnalysis_2Dmetals*” and “*VoronoiChargeAnalysis_2Dmetals*” directories house three tables each, summarizing the outcomes of charge population analyses conducted using Hirshfeld and Voronoi schemes for 2D noble metals on bare 4H-SiC, buffer layer and epitaxial graphene, respectively.

Table 1 provides a comprehensive overview of structural parameters, Hirshfeld charge magnitudes on the 2D metal layers, and various energy-related metrics, offering valuable insights into the electronic properties of the materials.

**Table 1:** Structural and energetic parameters for free-standing and substrate-supported 2D metals (2DM).

2DM	Substrate		Mean 2DM – substrate distance, Å	Mean M-M bond length, Å	Corrugation of 2DM, Å	Charge on 2DM, *e^−^*	*E*_int_^BSSE^, eV	Deformation energy of substrate, eV	Deformation energy of 2DM, eV	*E*_b_, eV
Ag	no	1 × 3 × 1	–	3.0097	0	0	–	–	–	–
		2 × 6 × 1	–	3.0097	0	0	–	–	–	–
	4H-SiC	1 × 3 × 1	2.1753	3.1217	0.0040	0.5280	7.875	0.820	1.186	5.870
		2 × 6 × 1	2.1735	3.1220	0.0025	2.1120	31.617	3.415	4.685	23.518
	BuL	1 × 3 × 1	2.7510	3.0792	0.0501	0.0900	−0.128	0.113	0.688	−0.929
		2 × 6 × 1	2.7535	3.0793	0.0526	0.3120	−0.364	0.400	2.711	−3.475
	MEG	1 × 3 × 1	2.7405	3.0457	0.0472	0.0880	−0.270	0.025	0.328	−0.622
		2 × 6 × 1	2.7380	3.0457	0.0470	0.3920	−1.125	0.086	1.280	−2.491
Au	no	1 × 3 × 1	–	3.0069	0	0	–	–	–	–
		2 × 6 × 1	–	3.0069	0	0	–	–	–	–
	4H-SiC	1 × 3 × 1	2.5975	3.1257	3.283 × 10^−4^	0.2220	5.9695	0.572	0.746	4.652
		2 × 6 × 1	2.5962	3.1256	8.386 × 10^−4^	0.8880	23.986	2.327	2.884	18.775
	BuL	1 × 3 × 1	2.9678	3.0829	0.0577	0.0240	−0.502	0.042	0.407	−0.952
		2 × 6 × 1	2.9676	3.0833	0.0595	0.0160	−1.716	0.186	1.576	−3.478
	MEG	1 × 3 × 1	2.9518	3.0481	0.0327	0.0340	−0.631	0.014	0.188	−0.833
		2 × 6 × 1	2.9453	3.0486	0.0404	0.1840	−2.584	0.024	0.726	−3.334
Ir	no	1 × 3 × 1	–	2.6710	0	0	–	–	–	–
		2 × 6 × 1	–	2.6711	0	0	–	–	–	–
	4H-SiC	1 × 3 × 1	2.3090	2.7488	0.0104	−0.686	17.607	0.907	6.168	10.532
		2 × 6 × 1	2.3114	2.7514	0.0050	−2.744	70.536	3.694	25.053	41.789
	BuL	1 × 3 × 1	2.2082	2.8115	0.0691	−0.662	12.346	3.012	6.561	2.7714
		2 × 6 × 1	2.2064	2.8225	0.0685	−2.672	49.862	11.896	26.945	11.021
	MEG	1 × 3 × 1	2.0727	2.6829	0.0067	−0.694	5.4260	0.531	5.295	−0.400
		2 × 6 × 1	2.0699	2.6789	0.0122	−2.800	22.586	2.291	21.664	−1.369
Os	no	1 × 3 × 1	–	2.6229	0	0	–	–	–	–
		2 × 6 × 1	–	2.6229	0	0	–	–	–	–
	4H-SiC	1 × 3 × 1	2.3746	2.7468	0.0261	−0.294	16.843	1.044	8.518	7.281
		2 × 6 × 1	2.3531	2.7174	0.0311	−1.176	65.406	3.962	32.236	29.208
	BuL	1 × 3 × 1	2.0838	2.6599	0.0031	−0.285	12.472	1.869	11.082	−0.480
		2 × 6 × 1	2.0845	2.6681	0.0019	−1.152	49.833	7.497	43.924	−1.587
	MEG	1 × 3 × 1	4.2370	2.5682	0.0181	−0.064	0.3700	0.056	6.399	−6.085
		2 × 6 × 1	4.2371	2.5682	0.0182	−0.428	1.4941	0.239	26.054	−24.799
Pd	no	1 × 3 × 1	–	2.6898	0	0	–	–	–	–
		2 × 6 × 1	–	2.6898	0	0	–	–	–	–
	4H-SiC	1 × 3 × 1	2.2849	3.1111	3.672 × 10^−4^	−0.114	16.320	0.941	5.970	9.409
		2 × 6 × 1	2.2846	3.1113	7.155 × 10^−4^	−0.456	64.484	3.825	24.586	36.073
	BuL	1 × 3 × 1	2.2288	2.7277	0.0444	0.0400	7.3748	1.885	4.522	0.968
		2 × 6 × 1	2.2292	2.7269	0.0445	0.1600	29.791	7.343	19.558	2.8902
	MEG	1 × 3 × 1	2.0937	3.0543	0.0106	0.0780	3.5083	0.095	4.966	−1.552
		2 × 6 × 1	2.0937	3.0544	0.0100	0.3280	13.572	0.454	20.604	−7.486
Pt	no	1 × 3 × 1	–	2.7877	0	0	–	–	–	–
		2 × 6 × 1	–	2.7877	0	0	–	–	–	–
	4H-SiC	1 × 3 × 1	2.3430	3.1114	7.987 × 10^−4^	−0.144	16.894	0.939	4.756	11.200
		2 × 6 × 1	2.3434	3.1124	0.0010	−0.576	66.453	3.776	19.712	42.965
	BuL	1 × 3 × 1	2.3045	2.9945	0.0234	0.0180	7.7723	2.459	4.084	1.229
		2 × 6 × 1	2.3068	2.9963	0.0223	0.0720	30.478	9.504	17.557	3.417
	MEG	1 × 3 × 1	2.2196	2.9173	0.0282	0.0460	2.5328	10.938	3.741	−12.15
		2 × 6 × 1	2.2168	2.9155	0.0235	0.2000	10.251	43.631	16.499	−49.88
Rh	no	1 × 3 × 1	–	2.5718	0	0	–	–	–	–
		2 × 6 × 1	–	2.5718	0	0	–	–	–	–
	4H-SiC	1 × 3 × 1	2.2300	2.7254	0.0024	−0.032	17.562	0.948	7.939	8.6751
		2 × 6 × 1	2.2253	2.7199	0.0025	−0.112	70.202	3.882	32.1	34.221
	BuL	1 × 3 × 1	2.1444	2.7680	0.0636	0.0800	12.945	2.683	8.119	2.143
		2 × 6 × 1	2.151	2.7139	0.055963	0.256	49.718	10.241	30.419	9.057
	MEG	1 × 3 × 1	1.9891	2.6603	0.0085	0.0940	7.7446	0.400	6.996	0.349
		2 × 6 × 1	1.9888	2.6564	0.0079	0.3600	31.965	1.846	28.515	1.604
Ru	no	1 × 3 × 1	–	2.5487	0	0	–	–	–	–
		2 × 6 × 1	–	2.5487	0	0	–	–	–	–
	4H-SiC	1 × 3 × 1	2.2229	2.7195	0.0281	0.1200	16.082	0.961	8.108	7.013
		2 × 6 × 1	2.2228	2.7190	0.0213	0.4960	64.569	3.944	32.358	28.267
	BuL	1 × 3 × 1	2.0277	2.5502	4.373 × 10^−4^	0.1580	14.063	1.470	11.499	1.094
		2 × 6 × 1	2.0287	2.5513	6.995 × 10^−4^	0.6480	56.236	5.845	45.488	4.9033
	MEG	1 × 3 × 1	1.9252	2.6195	0.0083	0.1880	11.210	0.066	11.241	−0.097
		2 × 6 × 1	1.9256	2.6176	0.0083	0.7520	44.407	0.294	44.395	−0.281

**Fig. 3:**
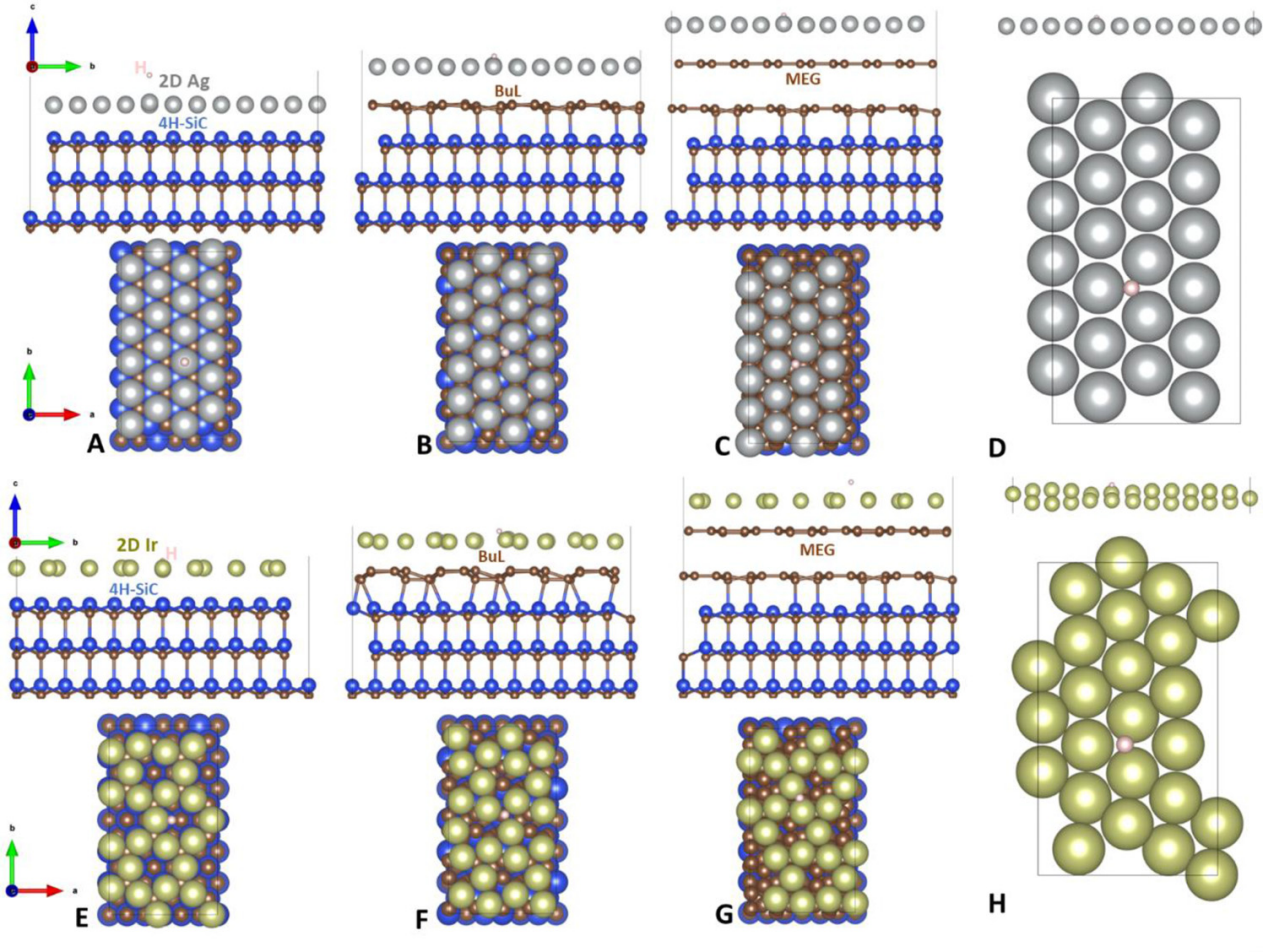
(Side and top views) Optimized atomic structures of substrate-supported (4H-SiC, BuL and MEG) and free-standing Ag (A–D) and Ir (E–H) monolayers after hydrogen adsorption, respectively. Blue, brown, gray, mustard green and whitish designate Si, C, Ag, and Ir atoms, respectively. Note: To enhance visualization, the top-view images of the optimized structures are presented in a space-filling representation using the VESTA program.

The fifth root directory, “*HydrogenAdsorption_2DMetals*” comprises four subfolders, each containing eight additional subfolders. These subfolders present atomic structures in top and side views of free-standing and substrate-supported 2D noble metals after hydrogen adsorption. Cartesian coordinates for these structures are also included. Notably, the hydrogen adsorption process was modeled using the (2 × 6 × 1) supercell model for both free-standing and substrate-supported 2D noble metals. Fig. 3 illustrates the optimized structures of 2D Ag and 2D Ir with adsorbed hydrogen species, while data for other metals are provided as supplemental files within this directory. Table 2 offers insights into the Gibbs free energy (*ΔG*_H*_) for hydrogen adsorption on both free-standing and substrate-supported 2D noble metals.

**Table 2:** Gibbs free energy (Δ*G*_H*_) for H adsorbed on free-standing and substrate-supported 2D noble metals. Note: As convergence has not been achieved for the H/2DOs/MEG structure using the vdW-BH functional, the Gibbs free energy for hydrogen adsorption remains unavailable for this particular configuration.

2DM	Δ*G*_H*_, eV			
	no substrate	4H-SiC	BuL	MEG
Ag	−0.079 (−0.087)	1.095	−2.025	−0.116
Au	1.365 (0.251)	0.499	−0.824	0.221
Ir	−2.407 (−2.463)	−0.401	−0.759	−0.486
Os	−3.895 (−3.758)	−2.269	−0.855	–
Pd	−0.902 (−1.338)	0.954	−0.798	−0.720
Pt	−0.784 (−1.234)	0.828	−0.342	−0.691
Rh	−1.631 (−2.252)	−1.189	−1.896	−0.545
Ru	−3.189 (−2.922)	−2.868	−1.152	−4.916

This meticulously structured dataset serves as a valuable resource for researchers and scientists seeking to explore the properties and behaviors of 2D noble metals in various configurations.

## 3 Experimental Design, Materials and Methods

The SIESTA code [2] was the platform for all DFT calculations. In each case, the vdW-BH functional was applied in conjunction with a double-ζ polarized (DZP) basis set [3]. Drawing from established expertise [4], [5], [6], [7], [8], the DZP basis set consistently yields satisfactory outcomes for the MEG/BuL/SiC system, capturing the nuanced interactions with noble metals.

The parameters were meticulously configured: an energy shift of 200 meV and a force tolerance of 0.02 eV/Å. In relaxation-type DFT calculations, the Brillouin zone was sampled using a 9 × 9 × 1 Monkhorst–Pack *k*-grid. Conversely, electronic and charge transfer property calculations employed a 24 × 24 × 1 *k*-point grid. The ATOM code [9] was employed to generate norm-conserving Troullier-Martins pseudopotentials for C, Si, noble metal, and H atoms. The electronic convergence tolerance was established at 10^—6^ eV, with the mesh cutoff energy for the integration grid consistently fixed at 400 Ry. Structure relaxation, encompassing both cartesian coordinates and lattice vectors, continued until reaching a force tolerance of 0.02 eV/Å. The maximum allowable atomic displacement was restricted to 0.05 Å. Additionally, the *PAO.SplitNorm* parameter was thoughtfully configured to 0.25, while both density matrix parameters, *DM.MixingWeight* and *DM.NumberPulay*, were deliberately assigned values of 0.05 and 10, respectively. The electronic structure calculations were conducted employing the standard diagonalization method (DIAGON) within SIESTA. To represent the electronic properties accurately, an electronic temperature of 50 meV was employed. Furthermore, the *WriteHirshfeldPop* and *WriteVoronoiPop* flags were activated to enable the calculation of the Hirshfeld and Voronoi “net” atomic populations, thus facilitating a comprehensive understanding of atomic interactions. It is important to emphasize that all results obtained under these parameter settings are valid solely at *T* = 0 K, shedding light on the intrinsic low-energy characteristics of metal-template systems.

As the substrate for SiC and the foundation for BuL and MEG formation, the Si-face 4H-SiC was employed. To emulate MEG, a 2 × 2 hexagonal graphene lattice was initially crafted on a √3 × √3R30° reconstructed SiC (0001) surface, a recognized model for epitaxial graphene on 4H-SiC (0001) [10], [11], [12]. Subsequently, BuL/SiC was modeled by removing the uppermost graphene layer from the EG structure. To establish the interface between the face-centered-cubic (fcc) unit cell of selected noble metals (Au, Ag, Pd, Pt, Rh, and Ir) and the hexagonally packed structures of SiC, BuL/SiC, and MEG/SiC, a transformation was initiated from the hexagonal √3 × √3R30°-model to an orthorhombic unit cell model (refer to the “*Substrates*” folder within “*Structure_2Dmetals*”). This transformation retained the hexagonal packaging while introducing the metal (111) layer onto the SiC, BuL, or MEG surface. The (1 × 3 × 1) metal (111) supercell harmonized effectively with the rectangular (2 × 2 × 1) supercell of SiC, BuL, or MEG. Notably, due to the hexagonal close-packed (hcp) crystal structure preference of Ru and Os, the (0001) 2D Os and (0001) 2D Ru layers were considered.

Ultimately, the unit cell was duplicated twice in the in-plane directions to encompass a single metal monolayer containing 24 atoms, which was placed onto a slab surface (referred to as the 2 × 6 × 1 model). This model, featuring nine Si-C bilayers, was utilized to explore the adhesion of the 2DM to the substrate and the catalytic activity of substrate-supported 2D metals. In pursuit of computational efficiency, the cartesian coordinates of the atomic positions within seven of the low-lying Si-C bilayers were deliberately held constant. To gain a deeper understanding of the substrate's impact on the properties of 2D metals, investigations of free-standing 2D metal layers were also conducted. To prevent undesired interactions between the slab and its periodic replica, a vacuum layer of 25 Å was added above the surface along the slab-normal direction.

The Multiwfn program [13] was employed to compute plane-averaged 1D CDD curves, and their visual representations were generated using MATLAB R2021b [14]. Additionally, Gaussian cube files containing charge density differences at each grid point were created using the GaussView 6 interface [15]. For the visualization of optimized structures and 3D CDD maps, the VESTA program [16] was utilized.

The corrugation of the 2DM layer was ascertained by calculating the difference between the maximum *z*-coordinate value for certain metals and the mean *z*-coordinate value for those metals. This metric served as an indicator of the planarity of the metal monolayer. Similarly, the disparity between the mean *z*-coordinate value for a certain metal layer and the mean *z*-coordinate value for the topmost layer of the substrate was considered as the average 2DM-support distance. The mean M–M bond length was determined through an analysis of the shortest M–M bonds within the 2DM layer.

To gauge the interaction strength within the 2DM/support system, the estimation of the Basis Set Superposition Error (BSSE)-corrected binding energy was carried out employing the following equations:

(1) $$E_{b}=E_{tot}^{2DM}+E_{tot}^{S}-E_{tot}^{2DM-S}+E_{tot}^{2DM^{ghost}}-E_{tot}^{2DM^{2DM-S}}+E_{tot}^{S^{ghost}}-E_{tot}^{S^{2DM-S}}$$

Herein, $E_{tot}^{S}$ denotes the complete energy of the isolated support (SiC, BuL or MEG), $E_{tot}^{2DM}$ signifies the total energy of the isolated 2DM layer, and $E_{tot}^{2DM-S}$ stands as the total energy of the 2DM/support system. The four remaining terms within Eq. (1) serve the purpose of estimating the BSSE correction energy. $E_{tot}^{2DM^{2DM-S}}$ and $E_{tot}^{S^{2DM-S}}$ correspond to the total energies of the 2DM layer and the support in the relaxed configuration of the 2DM/support system, while $E_{tot}^{2DM^{ghost}}$ and $E_{tot}^{S^{ghost}}$ represent the total energies of the 2DM layer and support in the relaxed structure of the 2DM/support system, incorporating ghost atoms.

By rearranging Eq. (1) and introducing three novel terms, namely the interaction energy component $E_{int}^{BSSE}$, and deformation energies $E_{def}^{2DM},\;E_{def}^{S}$, the following expression can be formulated:

(2) $$\left\{ \begin{array}{c} E_{b}=E_{tot}^{2DM^{ghost}+}E_{tot}^{S^{ghost}}-E_{tot}^{2DM-S}-\left(E_{tot}^{2DM^{2DM-S}}-E_{tot}^{2DM}\right)-\left(E_{tot}^{S^{2DM-S}}-E_{tot}^{S}\right)\\ E_{b}=E_{int}^{BSSE}-E_{def}^{2DM}-E_{def}^{S} \end{array}\right.$$

From a physical perspective, these deformation energies serve the purpose of estimating the energy penalties incurred in accommodating 2DM layers on a supporting substrate.

To evaluate the influence of the substrate on the catalytic performance of 2DM layers, modeling of the Volmer reaction, the preliminary step in the hydrogen evolution reaction, was performed on the surfaces of 2DM, 2DM/SiC, 2DM/BuL, and 2DM/MEG. This modeling involved the estimation of the Gibbs free energy of hydrogen adsorption (Δ*G*_H*_), calculated using the following equation [17]:

(3) $$\Delta G_{H*}=\Delta E_{H*}+0.24\;\text{eV}$$

In Eq. (3), Δ*E*_H*_ signifies the adsorption energy of hydrogen, while the term 0.24 eV accounts for the energy variations in zero-point energy (ZPE) and entropy between gaseous H and absorbed H.

## Limitations

Not applicable.

## Ethics Statement

This research adheres to the ethical standards necessary for publication in Data in Brief, as it does not involve any human or animal subjects.

## CRediT authorship contribution statement

**Ivan Shtepliuk:** Investigation, Writing – original draft, Methodology, Conceptualization, Writing – review & editing.

## Data Availability

Free-standing and substrate-supported 2D noble metals (Original data) (Mendeley Data)
